# Achieving Universal Health Coverage and Health-Related Sustainable Developmental Goals in Nepal through Traditional, Complementary and Integrative Medicine

**DOI:** 10.31729/jnma.9194

**Published:** 2025-08-31

**Authors:** Bibek Giri, Vijay Kumar Chattu

**Affiliations:** 1 Research Institute for Collaborative Development, Kathmandu, Nepal; 2 Department of Occupational Science & Occupational Therapy, Temerty Faculty of Medicine, University of Toronto, Toronto, Canada

**Keywords:** *traditional*, *complementary and alternative medicine*, *nepal*, *healthcare system*, *safety*, *rural health*, *community health*

## Abstract

Nepal faces significant challenges in providing equitable healthcare due to limited infrastructure, high cost, and geographic barriers. Integrating Traditional, complementary and integrative medicine into the Nepalese healthcare system could address these challenges by offering affordable, culturally relevant, and holistic care. The analysis identifies both advantages, such as cost-effectiveness and cultural acceptance, and challenges, including regulation, standardization, and professional training needs. Recommendations include incorporating TCIM into primary healthcare, providing education and training for healthcare providers, establishing guidelines and regulatory frameworks, conducting research on safety and efficacy, and promoting community engagement. Integrating TCIM could strengthen healthcare delivery, reduce disparities, and support Nepal’s progress toward universal health coverage and sustainable development goals.

## INTRODUCTION

Nepal’s healthcare system struggles with accessibility, cost, and cultural diversity, leaving rural populations underserved.^1^ Traditional, Complementary, and Integrative Medicine (TCIM) is widely practiced, offering holistic, affordable, and culturally acceptable care.^2^ This study explores how TCIM can complement conventional medicine to help Nepal achieve universal health coverage (UHC) by 2030.^4^ By assessing TCIM’s benefits and integration challenges, this research aims to provide actionable recommendations for policy, education, and practice. Integrating TCIM could bridge therapeutic gaps, enhance equity, and build a more resilient, inclusive healthcare system that respects Nepal’s cultural heritage.

## RELEVANCE OF TCIM IN NEPAL

According to Bhattarai et al.,^5^ in Nepal, People often postpone seeking medical help until their condition deteriorates. This delay can stem from various reasons: they may grow accustomed to their symptoms, mistrust health services, prefer alternative treatments, rely on self-care, or perceive modern healthcare as too costly.^5^ The first-ever World Health Organization (WHO) Traditional Global Summit 2023, held in Gujarat, India, highlighted the scope of TCIM in improving the progress towards achieving UHC and health-related SDGs.^6^ Thus, combining TCIM into healthcare provision in Nepal could tackle problems such as inadequate infrastructure, therapeutic gaps, and knowledge gaps. Incorporating TCIM into Nepal’s healthcare system could surmount challenges in healthcare provision, enhance the availability of low-cost and holistic healthcare services, deal with the absence of infrastructure in rural areas, and offer a more complete and patient-focused approach to healthcare in Nepal. As highlighted by Pandey, Nepal should frequently review how diseases affect its population, measure how well interventions work and spend more money on cheap and effective interventions.^4^ Thus, adding TCIM practices into Nepal’s healthcare system can assist in reducing costs, increasing community acceptance and addressing healthcare provision challenges in steep and mountainous areas where resources are insufficient.

## TCIM AS A POTENTIAL CONTRIBUTOR TO THE EXISTING HEALTHCARE

TCIM could play a crucial role in addressing healthcare challenges in Nepal. It offers various healing methods that can either complement or replace conventional biomedicine.^7^ TCIM offers a unique opportunity to strengthen healthcare in Nepal .^7^ With its deep cultural roots and a wealth of traditional practices passed down through generations, Nepal is in a prime position to blend these time-honored approaches with modern medical care, creating a more holistic and accessible system for its people. For example, practices like Ayurveda and traditional medicine are widely accepted in many cultures, including neighboring India.^8^ This acceptance could pave the way for Nepal to integrate TCIM into its healthcare system, providing affordable treatment options for those who can’t afford modern medical care.

A study by Kruk et al. highlights that improving the quality of healthcare services could prevent about 57% of avoidable deaths, while increasing service utilization could prevent the remaining 43%,^9^ By incorporating TCIM, Nepal could enhance healthcare delivery, especially in remote, mountainous regions with limited infrastructure. TCIM can offer more treatment choices, reduce costs, and improve the quality of holistic care. Integrating TCIM into Nepal’s healthcare system can provide a more holistic and patient-centered approach, expanding healthcare options and increasing patient satisfaction and engagement in their own care.^10^

## TCIM AS A BRIDE TO ADDRESS INEQUALITIES

The mental health difficulties of migrant workers worldwide TCIM could help overcome healthcare delivery obstacles in Nepal, especially in hilly and mountainous areas with inadequate infrastructure.^11^ Many authors have highlighted that some people use health services and protect themselves from financial risk more than others, depending on where they live, how much money they have, how much education they have, and what ethnic group they belong to. They also emphasized that some people may be excluded from society and that current health policies do not address this problem well enough.^12,13^ This is particularly important in areas where conventional healthcare options are limited or inaccessible due to geographical barriers. Moreover, evaluating the awareness, perception, and application of TCIM among Nepalese people in remote areas can reveal their healthcare habits and assist in finding the advantages and difficulties of incorporating TCIM into the healthcare system in Nepal.^11^ The effective integration of TCIM into the healthcare system in Nepal relies on doctors and other health professionals’ awareness of TCIM.^14^ They should be educated about TCIM modalities, their safety and effectiveness, and how they can complement conventional treatments. Moreover, TCIM incorporation into Nepal’s healthcare system could boost the ability to provide comprehensive services and bridge therapeutic gaps, offering more healthcare alternatives for the people. By supporting TCIM practices, healthcare providers and managers can accommodate the needs and views of the Nepalese people, thus enhancing patient Satisfaction and involvement in their healthcare journey.

## TCIM USE IN NEPAL AND EVIDENCE FROM OTHER COUNTRIES

Furthermore, TCIM incorporation can also assist in addressing the scarce data and information on medical approaches towards TCIM in Nepal, creating a basis for more research and comprehension of its efficacy and security in the Nepalese setting. Various examples of TCIM applications in developing countries demonstrate the possible advantages and difficulties of incorporating TCIM into healthcare systems. For instance, a study in India revealed that applying traditional Ayurvedic medicine and conventional treatments resulted in better outcomes for patients with chronic conditions such as high blood pressure and diabetes.^15,16^ Another study done in rural China discovered that incorporating traditional Chinese medicine into primary healthcare services led to decreased healthcare expenses and improved patient satisfaction.^17,18^ Nepal, situated in the Hindu Kush Himalayan (HKH) region, has a deep-rooted tradition of using natural medicines, cherished and passed down through generations as part of its community’s way of life.^19^ In Nepal the utilization of TCIM is high in general population. According to the Kadayat et al.,^20^ almost 55.69% people were using more than one type of TCIM. Where patients with chronic disease conditions are more likely to use TCIM, especially Ayurveda.^21^ Most of the people in Nepal perceived TCIM as an affordable, effective and free from side effects.

## ADVANTAGES AND DISADVANTAGES OF INCORPORATING TCIM IN NEPAL

TCIM encompasses various medical and healthcare practices that are not part of mainstream medicine, and implementation of TCIM in Nepal has pros and cons. On the positive side, integrating TCIM into the healthcare system can provide additional treatment options for patients, especially in areas where conventional healthcare services are limited or inaccessible.^10^ This can lead to increased patient satisfaction and empowerment as they have more choices in their healthcare. TCIM implementation in Nepal could offer more healthcare options to people, allowing them to have more preferences for their health and wellness. TCIM aims to heal the entire person, encompassing their bodily, mental, and emotional health. This method could result in enhanced health outcomes and a higher overall quality of life for people in Nepal^22^. TCIM practices are often based on traditional and native cultures. TCIM implementation in Nepal would recognize and respect the country’s cultural legacy, enabling people to use healthcare practices that are culturally suitable and known to them.

On the other hand, there may be challenges in the regulation and standardization of TCIM practices to ensure safety and effectiveness. In the Absence of regulation and standardization, TCIM practices can differ significantly in their safety, efficacy, and quality^23^. This absence of regulation and standardization can create dangers for people wanting TCIM treatments in Nepal. Many TCIM practices have not been thoroughly researched or supported by solid scientific evidence^24^. Additionally, TCIM integration into the healthcare system may need more resources and training for healthcare providers to ensure they are sufficiently prepared to offer TCIM therapies.

## APPROACHES AND RECOMMENDATIONS FOR INCORPORATING TCIM

Many people in Nepal use complementary and alternative medicine to improve their health and well-being, and to address social and economic challenges. Integrating TCIM into Nepal’s healthcare system can be achieved through practical steps like incorporating TCIM services in primary healthcare centers, especially in rural areas, and including TCIM concepts in medical and nursing education. Engaging with communities through training sessions, lectures, and community service activities can help people make informed healthcare choices. To ensure the quality and effectiveness of TCIM in Nepal’s healthcare sector, it’s important to establish rules and guidelines. TCIM providers should have the necessary permits and oversight to maintain professional standards and protect public health. Research projects should be conducted to evaluate the quality, safety, and affordability of TCIM therapies for the Nepalese population. It’s also crucial to involve and educate the community about the benefits and drawbacks of TCIM. Providing reliable information on various TCIM treatments can increase public awareness and understanding. TCIM is already part of the healthcare systems in countries like China, India, and South Korea, showing its potential for successful integration in Nepal^25^. By enhancing healthcare availability, supplementing existing methods, reducing disparities in medical practices, and offering cost-effective, long-term solutions, TCIM can improve healthcare delivery and health outcomes in Nepal. This is especially important for remote and mountainous regions with inadequate infrastructure.^11^

Effective integration of TCIM into mainstream healthcare requires good communication and collaboration between conventional healthcare providers and TCIM practitioners. Traditional practitioners bring valuable knowledge of herbal remedies, acupuncture, and other complementary therapies^26^, while modern healthcare providers offer advanced medical treatments and technologies.^27^ This teamwork helps identify potential interactions between traditional and modern treatments, ensuring patients receive safe and effective care. Additionally, joint efforts in education and training can equip healthcare professionals with a broader understanding of various treatment options, fostering mutual respect and better communication. Ultimately, this integrated approach enhances patient trust, promotes holistic well-being, and ensures all aspects of a patient’s health are addressed safely and effectively.

However, implementing TCIM in Nepal faces several challenges. The infrastructure in rural areas is often underdeveloped, lacking the necessary equipment and resources to support TCIM practices.^28^ Health education is another issue, as many healthcare professionals need training in TCIM, requiring educational reforms to include these concepts in medical and nursing curricula. Financial constraints limit funding for TCIM research and implementation. Additionally, regulatory and standardization issues need to be addressed to ensure the quality and safety of TCIM practices and products. Public awareness and acceptance of TCIM can be low, especially in urban areas where modern medicine is more prevalent.^29^ Finally, Nepal’s rugged terrain poses geographical challenges, making it difficult to distribute TCIM services and products to remote communities. Overcoming these challenges will require coordinated efforts involving government support, educational reforms, infrastructure development, and public awareness campaigns.

This study is a narrative review and relies on previously published literature, which may be limited in scope, quality, and availability, particularly regarding TCIM practices in Nepal. Evidence from rural and remote areas is scarce, and many studies focus on neighboring countries, which may limit direct generalizability. Additionally, variations in TCIM practices, regulatory frameworks, and cultural acceptance may affect the applicability of findings. Finally, language restrictions to English publications could have excluded relevant local studies published in Nepali.

## CONCLUSIONS

Integrating TCIM into Nepal’s healthcare system could make a big difference, especially for people in remote areas. It would provide affordable and comprehensive care, helping to reduce healthcare inequality. TCIM offers cost-effective treatments, improves services, gives patients more choices, and increases their satisfaction. By using TCIM practices, Nepal can address the lack of healthcare infrastructure in remote areas and move closer to UHC and health-related SDGs. Educating healthcare providers about TCIM can help patients feel more comfortable discussing these practices, leading to more holistic care.

For TCIM to be successfully integrated into mainstream healthcare, there needs to be good communication and collaboration between healthcare providers and TCIM practitioners. It’s important to develop guidelines, protocols, and regulatory frameworks. The future of TCIM in Nepal’s healthcare sector looks promising for tackling challenges and improving healthcare delivery. By offering education and training programs, establishing guidelines, creating a regulatory framework, supporting research, and advocating for policy changes, Nepal can effectively use TCIM therapies.

## References

[1] Adhikari B, Mishra SR, Schwarz R (2022). Transforming Nepal's Primary Health Care Delivery System in Global Health Era: Addressing Historical and Current Implementation Challenges.. Global Health..

[2] Bhusal S, Paudel R, Gaihre M, Paudel K, Adhikari TB, Pradhan PMS (2021). Health Literacy and Associated Factors Among Undergraduates: A University-Based Cross-Sectional Study in Nepal.. PLOS Glob Public Health..

[3] World Health Organization (2021). Universal Health Coverage..

[4] Pandey AR (2023). Health Financing Reforms in the Quest for Universal Health Coverage: Challenges and Opportunities in the Context of Nepal.. J Glob Health..

[5] Bhattarai S, Parajuli SB, Rayamajhi RB, Paudel IS, Jha N (2015). Health Seeking Behavior and Utilization of Health Care Services in Eastern Hilly Region of Nepal.. J Coll Med Sci-Nepal..

[6] World Health Organization (2023). Global partners commit to advance evidence-based traditional, complementary and integrative medicine..

[7] Najibi SM, Sarikhani Y, Hajimonfarednejad M, Nimrouzi M, Hashempur MH (2025). A Scoping Review of the Barriers and Facilitators in the Use of Traditional, Complementary, and Integrative Medicine: Insights for Health Policy Development.. J Health Popul Nutr..

[8] Chandwani R, De R (2015). The Institutional Dynamics Perspective of Ict for Health Initiatives in India.. Impact of Information Society Research in the Global South..

[9] Kruk ME, Gage AD, Joseph NT, Danaei G, Garca-Sais S, Salomon JA (2018). Mortality Due to Low-Quality Health Systems in the Universal Health Coverage Era: A Systematic Analysis of Amenable Deaths in 137 Countries.. Lancet..

[10] Mahmoodi MR, Shafian S, Alinaghizade MS (2023). The Effectiveness of Teaching Complementary and Alternative Medicine Based on the Components of Theory of Planned Behavior on Nutrition Students: Multicenter Research Study.. BMC Med Educ..

[11] Kafle PP, Pant P, Dhakal N (2021). Assessment of Health Seeking Behavior Regarding Complementary and Alternative Medicine in Solukhumbu District, Nepal.. Nepal Med Coll J..

[12] Pandey AR, Ojha B, Shrestha N, Maskey J, Sharma D, Godwin P (2021). Progress in Reducing Inequalities in Reproductive, Maternal, Newborn and Child Health Services in Nepal.. J Nepal Health Res Counc..

[13] Thapa AK, Pandey AR (2020). National and provincial estimates of catastrophic health expenditure and its determinants in Nepal.. J Nepal Health Res Counc..

[14] Ameade EPK, Amalba A, Helegbe GK, Mohammed BS (2016). Medical Students' Knowledge and Attitude Towards Complementary and Alternative Medicine - a Survey in Ghana.. J Tradit Complement Med..

[15] Lodha R, Bagga A (2000). Traditional Indian Systems of Medicine.. Ann Acad Med Singap..

[16] Gordon A, Buch Z, Baute V, Coeytaux R (2019). Use of Ayurveda in the Treatment of Type 2 Diabetes Mellitus.. Glob Adv Health Med..

[17] Chung VCH, Ma PHX, Wang HHX, Wang JJ, Hong LC, Wei X (2013). Integrating Traditional Chinese Medicine Services in Community Health Centers: Insights into Utilization Patterns in the Pearl River Region of China.. Evid Based Complement Alternat Med..

[18] Meng F, Ji Z, Song F, Bai T, Fan X, Wang D (2020). Patients' Familiarity with, Trust in and Willingness to Pay for Traditional Chinese Medicine in Chinese Community Health Care Centres.. Eur J Integr Med..

[19] Tali BA, Khuroo AA, Nawchoo IA, Ganie AH (2019). Prioritizing conservation of medicinal flora in the Himalayan biodiversity hotspot: an integrated ecological and socioeconomic approach.. Environ Conserv..

[20] Kadayat TM, Bist G, Parajuli A, Karki R, Kaundinnyayana A, Dhami N (2012). Patterns and perception of complementary and alternative medicine use by patients in western Nepal.. J Public Health (Berl.)..

[21] Choi SJ, Kunwor SK, Im HB, Hwang JH, Choi D, Han D (2022). Traditional and Complementary Medicine Use Among Cancer Patients in Nepal: A Cross-Sectional Survey.. BMC Complement Med Ther..

[22] Poudel M, Pandit M, Kumar Shrivastava A, Paudel Chhetri A, Pandey D, Pandey B (2023). Knowledge, Attitude and Perception of Undergraduate Health Science Students Towards Complementary and Alternative Medicine: A Cross-Sectional Study in Nepal.. Annapurna J H Sci..

[23] White A, Boon H, Alraek T, Lewith G, Liu JP, Norheim AJ (2014). Reducing the Risk of Complementary and Alternative Medicine (cam): Challenges and Priorities.. Eur J Integr Med..

[24] Clauson VA (2010). Current Issues Regarding Complementary and Alternative Medicine (CAM) in the United States: Part 1: The Widespread Use of CAM and the Need for Better-Informed Health Care Professionals to Provide Patient Counseling.. P T..

[25] Mbada CE, Adeyemi TL, Adedoyin RA, Badmus HD, Awotidebe TO, Arije OO (2015). Prevalence and Modes of Complementary and Alternative Medicine Use Among Peasant Farmers with Musculoskeletal Pain in a Rural Community in South-Western Nigeria.. BMC Complement Altern Med..

[26] World Health Organization (2023). Traditional medicine has a long history of contributing to conventional medicine and continues to hold promise..

[27] Yadav S (2024). Transformative Frontiers: A Comprehensive Review of Emerging Technologies in Modern Healthcare.. Cureus..

[28] Kapur DR (2024). Lack of Infrastructure, Amenities and Facilities: Impediment in Progression of Rural Communities.. IJMH..

[29] Motoo Y, Yukawa K, Hisamura K, Arai I (2021). Physician Perspectives on Traditional, Complementary, and Integrative Medicine and the National Evidence-Based Japanese Integrative Medicine Information Website: A Mixed-Method Study. Integr Med Res.

